# Hospital Finance in 1917

**Published:** 1918-04-20

**Authors:** 


### HOSPITAL FINANCE IN 1917.

Experience shows that a maximum price order generally has the effect of making the minimum and maximum prices the same, regardless of quality, with the result, as one instance, that all chocolates, whether good, bad, or indifferent, are now four shillings a pound. The effect of fixing a date for the publication of the accounts of hospitals, as is in effect done in London by the requirements of the Central Funds, apparently results in everybody aiming to produce their figures as near that date as possible. In the provinces, where there is no such compulsion, and a report can appear as late as the executive chooses or the governors will allow, the latitude results in many reports appearing long before London hospitals have made any sign of statistical life. Promptitude in the publication of a report must have a good effect upon the supporters of a charity, and if an important organisation, such as that of the Manchester Royal Infirmary, can present its governors with an account of its stewardship for the past year as early as January 22, it is difficult to excuse the lack of celerity on the part of other large provincial and metropolitan hospitals.

When the war broke out voluntary hospital committees were fearful that they were in for a bad financial time. There seemed every prospect of prices of daily necessaries being seriously increased and of revenues falling because of the diversion of public generosity into other channels. Certainly the first prediction has come true to an extent which few dreamed likely, but the second prophecy has fortunately failed. Purse-strings have been unloosed, people who never gave before have acquired giving habits, and the interest in the sick and the suffering induced by the hospitals for the wounded has extended practical sympathy to our established institutions. Many a hospital formerly suffering from a chronic yearly deficit has been put upon its legs, and many a moderately prosperous institution has been able to lay aside for a rainy day or for the extension of its activities.

From the income and expenditure accounts for 1917 that have reached us to date there is every indication that the general financial position is sound. Here and there we find an adverse balance, but never so crushing as to occasion alarm. The extraordinary activity of the committee and secretary of King Edward VII.'s Hospital, Cardiff, has resulted in a credit balance for the year of £2,164 as compared with a balance on the wrong side at the end of 1916 of £4,195. In Manchester the Royal Infirmary, with its substantial expenditure of £89,123, has failed to pay its way by £1,394, a result which, if annoying to Mr. Hazell and his committee, is, having regard to the extent of the undertaking, almost adventitious in its nature. Another Lancashire institution, the Blackburn and East Lancashire Royal Infirmary, has had better fortune, having spent but £12,067 out of its income of £14,301, but starting the year with an adverse balance belonging to 1916 of £3,378.

